# Computational and spectroscopic analysis of interaction between food colorant citrus red 2 and human serum albumin

**DOI:** 10.1038/s41598-018-38240-9

**Published:** 2019-02-07

**Authors:** Di Wu, Jinqiu Wang, Dayu Liu, Yin Zhang, Xia Hu

**Affiliations:** Key Laboratory of Meat Processing of Sichuan, College of Pharmacy and Biological Engineering, Chengdu University, Chengdu, 610106 China

## Abstract

The main aim of this work was to gain insight into the binding properties between a food colorant, citrus red 2 (CR), and human serum albumin (HSA), which is the predominant protein in blood plasma. Here, computer simulations and multiple spectroscopies were applied to predict and characterize the interaction between CR and HSA. Docking and molecular dynamics presented a stable binding configuration with low fluctuations. Fluorescence spectroscopy and lifetime results suggested that the CR–HSA combination undergoes static quenching mechanism with binding constant of 10^5^ L/mol. Displacement analysis showed the binding of CR at site I of HSA, which agrees with the docking results. The binding process occured spontaneously and was mainly driven by electrostatic interactions. Synchronous fluorescence and circular dichroism measurements demonstrate the changes in the microenvironment residues and *α*-helix contents of HSA induced by CR. The computational and experimental techniques are complementary to clearly understand the food colorant transportation and bioaccumulative toxicity in the human body.

## Introduction

As food colorants are ubiquitously used to imitate original and colorful food produce, their management and application have become key elements of scientific concern in recent years. Citrus red 2 (CR) is a synthetic azo colorant, which has been permitted by the United States Food and Drug Administration since 1956 and is mainly used on the skin of oranges^1^. However, as an azo dye, CR can be readily reduced to form aromatic amines and have potential health risks or a long-term cumulative toxicity such as genotoxicity, neurotoxicity, and carcinogenicity^2^. Over the last few decades, many countries have banned the use of CR as an additive in some foodstuff. However, making oranges appear visually pleasing is also important to consumers. Consequently, this food colorant is still enriched in some orange pulp and juice.

CR can easily enter the bloodstream after gastrointestinal absorption and then associates with physiologically macromolecules during transport^3^. Studies on food additives and biological macromolecules are concerned with their potential influences on human health. The characterization of interaction between CR and these macromolecules, such as plasma proteins, can provide a fresh insight to assess the safety of the food colorant.

Human serum albumin (HSA) constitutes approximately 50% of the proteins present in the plasma^4^, ranging from 33 g/L to 52 g/L in normal individuals^5^, and is involved in the distribution, clearance, and elimination of food additives. In addition, when HSA transports endogenous and exogenous substances in body fluids, the blood osmotic pressure and pH are maintained at a normal level^6^. Moreover, the primary ligand-binding regions are Sudlow’s site I and site II on HSA at its homologous *α*-helical domains^7–9^. Each site contains hydrophobic cavities which allow HSA to accommodate and transport various molecules^10,11^. If CR as a ligand binds to HSA, its solubility might increase, the molecular aggregates could decrease, and the half-life of CR would be extended. Therefore, we focus on investigating the binding properties between CR and HSA, which further clarifies the colorant–protein binding mechanism underlying the absorption of the synthetic food additives.

In this paper, the interaction between CR and HSA is systematically analyzed based on the computational and multi-spectroscopic technologies. Molecular docking and molecular dynamics (MD) were applied to theoretically assess the binding mode and stability of the combination. Fluorescence titration, synchronous fluorescence, and circular dichroism (CD) spectroscopy were utilized to clarify the binding mechanism, binding affinity, binding site, and conformational changes of HSA due to CR aggression. This work aims to establish a framework that can illuminate the binding properties of CR to HSA and thereby provide information on the safety of some similar synthetic food colorants.

## Results and Discussion

### Molecular docking simulations

**Figure 1:**
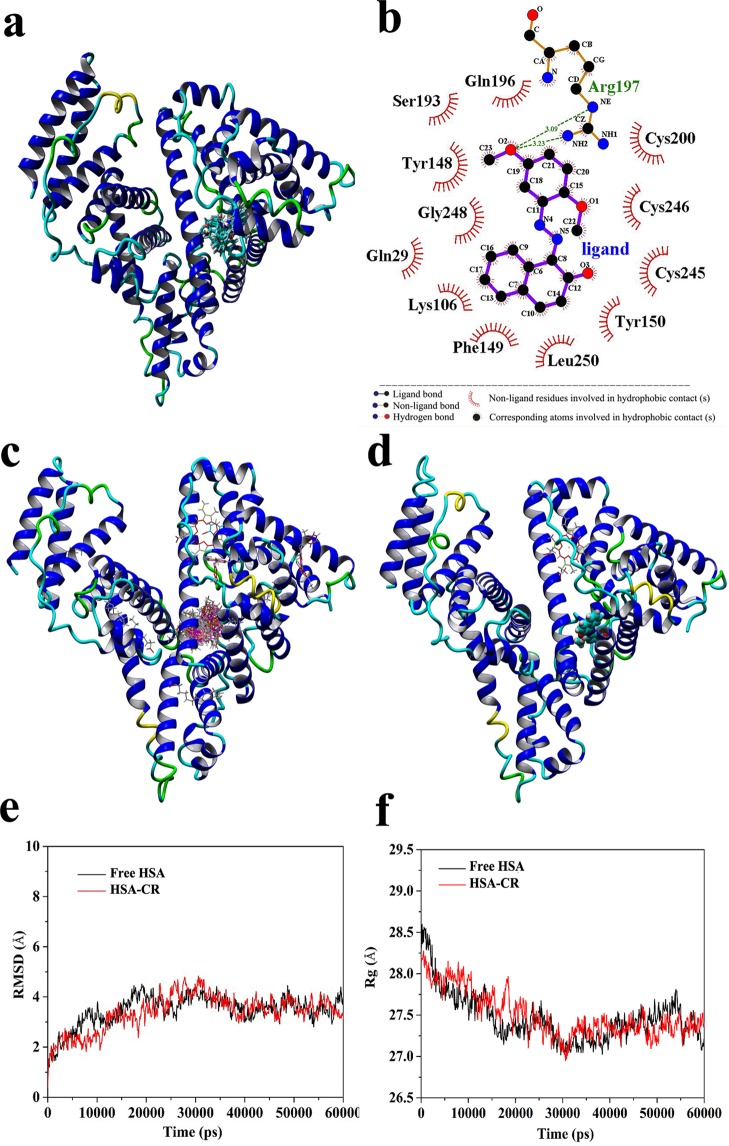
(**a**) 3D image of the cluster analysis for the first 15 conformations of CR binding to free HSA as generated by YASARA v17.4.17 model docking. (**b**) Schematic representation of the optimal conformation with interactions generated by LIGPLOT. (**c**) The 100 docking conformations of CR on HSA–hemin–myristate complex. (**d**) Optimal docking conformation of CR–heme:HSA. (**e**) Plot of root-mean-square deviation (RMSD) versus time in the MD simulation. (**f**) Graph of the radius of gyration (Rg) versus time in the MD simulation progress.

Automated docking simulations have been widely used in the initial procedures of active molecular development and the prediction of biomolecular complexes in structural or functional analysis^12–14^. For the theoretical prediction of possible realistic CR–HSA complex model, the YASARA strategy was performed to separately search the whole structure of different receptors (free HSA, receptor 1; HSA complexed with heme, receptor 2; and HSA complexed with myristic acid and hemin, receptor 3) and simultaneously optimize the conformations of the residues that build up the walls of the potential binding sites^15,16^. Crystallographic researches revealed that HSA is composed of three *α*-helical domains at different sites (sites I, II, and III located at subdomains IIA, IIIA, and IB, respectively) and is further separated into six-helix and four-helix subdomains^17,18^. After the process of docking calculation, the top 15 docking conformations of CR with free HSA were in the same cluster as displayed in Fig. 1a (2D rank one conformation was exhibited using the LIGPLOT program in Fig. 1b). The most important regions of CR in HSA are situated in the hydrophobic cavity of site I. As HSA is the primary fatty acid/heme-binding protein in extracellular fluids, the heme-binding cleft and Sudlow’s site I are functionally linked in their crystal textures^19–22^. The predicted binding position of CR on HSA was also confirmed by the relative visualizations of CR with receptors 2 and 3. Figure 1c shows all docking conformations (100 in total) of CR on the HSA–hemin–myristate complex. Moreover, 98% flexible CR was ultimately set at the site I of HSA. CR–heme:HSA has a similar optimal docking conformation (Fig. 1d). Based on the docking simulation results, the authentic binding orientation of CR is on the pocket of site I. As described in the 2D-partial map (Fig. 1b), the optimum pose in first of the class with 8.31 kcal/mol binding energy of CR at HSA encircled by several hydrophobic residues such as Gln29, Gln196, Ser193, Tyr148, Tyr150, Gly248, Lys106, Phe149, Leu250, Cys200, Cys245, and Cys246. The CR–HSA complex was stabilized by the hydrogen bonding interactions between the same oxygen atom of CR and two hydrogen atoms in the terminal amino group and the saturated imino group of Arg197 residue on HSA with lengths of 3.09 and 3.23 Å, respectively (Fig. 1b). This predicted observation promotes the structural basis and visual understanding of the binding mechanism.

### Molecular dynamics trajectory analysis

MD simulations were carried out under physiological conditions simulated to show the natural dynamics of CR–HSA system on different timescales. To evaluate the stability of binding, the root-mean-square deviation (RMSD) was figured out in the MD process to estimate the structural movement from the atomic fluctuation and the initial coordinates^23^. The RMSD values of heavy atoms for the free HSA and CR–HSA systems versus the simulation time are shown in Fig. 1c. The RMSD values of the MD trajectory were achieved from 0 ps to 60,000 ps. The system values were increased over time, reaching a plateau at approximately 30,000 ps. Furthermore, within the limits of 0–30,000 ps, the RMSD value was 3.37 Å in average, which was slightly smaller than that of free protein (3.51 Å). This finding implies that when the CR–HSA complex is fully shaped, the structure of the whole system would be slightly stable compared with that of free HSA. Furthermore, the radius of gyration (Rg) values, defined as the distribution of atoms around a given protein axis, were analyzed to assess the receptor integrity and the stability of the backbone atoms in the system^24^. If the system collapses, thereby the system expanding or decreasing, Rg values will be increased. Figure 1d represents that the Rg values of both free HSA and CR–HSA initially decreased and achieved equilibrium at approximately 32,000 ps in the complex system. The overall Rg values of CR–HSA did not substantially change compared with those before binding. The average Rg values were 27.61 and 27.57 Å for free HSA and CR–HSA, respectively. This finding indicates the optimal combination structure of the CR–HSA complex assumes a relatively stabilized binding position with low atomic fluctuations, which exhibited a better stabilization than free HSA.

### Fluorescence quenching mechanism

**Figure 2:**
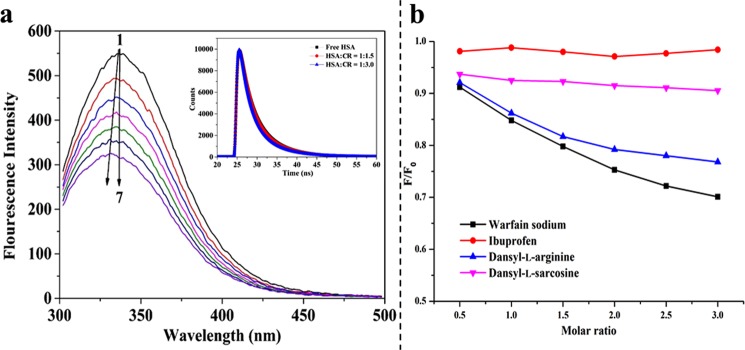
(**a**) Effect of CR on the fluorescence spectra of HSA (pH 7.4, *T* = 298 K and *λ*_em_ = 280 nm). (1–7) 2 µM HSA in the presence of 0, 1, 2, 3, 4, 5, and 6 µM CR, respectively. Inset: Time-resolved fluorescence lifetime of HSA in the absence and presence of various concentrations of CR. (**b**) Curves of fluorescence quenching of CR–HSA system in the presence of probes.

Quenching mechanism can be assorted as static, dynamic, and their combinations^25^. The combinations between the receptor and ligand can be illuminated by temperature dependent and time-resolved fluorescence measurements^12,26^. Figure 2(a) displays the influence of CR on the quenching of HSA fluorescence. As shown, an apparent blue shift can be observed at 338 nm, which is the typical fluorescence peak of HSA, suggesting that CR exerted an evident effect on the microenvironment of HSA residues. The quenching constants at different temperatures were determined by the Stern–Volmer equation:

1 $${F}_{0}/F=1+{K}_{SV}[Q]$$

where *F*_0_ and *F* are the fluorescence emission intensities with and without CR, respectively; *K*_sv_ is the Stern-Volmer quenching constant can be determined using the linear regression of the plot of *F*_0_/*F* against the concentration of CR [*Q*]. In the present system, *K*_SV_ values decrease with increasing temperature (Table 1), which indicates that the quenching of HSA by CR might be initiated by complex formation. In other words, specific ground state complexation is responsible for the quenching rather than dynamic collision^27^. Furthermore, this finding was verified through time-resolved fluorescence spectra (Fig. 1a inset) based on the principles of fluorescence spectroscopy^28^. Decay lifetime is nearly unaffected in ground-state quenching, whereas it decreases in excited-state quenching processes. As shown in Fig. 1a (inset), the effect of CR to HSA is negligible on the changes of fluorescence lifetime. The time region between the vertical lines was used for fitting a bi-exponential distribution to the measured and the fluorescence lifetime on vaerage via tail-fitting method^12,29^:

2 $$\tau =\sum {\alpha }_{i}{\tau }_{i}$$

**Table 1:** Stern–Volmer constants *K*_SV_, binding constants *K*_a_, and thermodynamic parameters for the CR–HSA system at different temperatures.

*T* (K)	*K*_SV_ × 10^5^ (L/mol)	*K*_a_ × 10^5^ (L/mol)	*n*	Δ*G* (kJ/mol)	Δ*H* (kJ/mol)	Δ*S* (J/mol/K)
298	1.26 ± 0.03	1.14 ± 0.03	1.00 ± 0.05	−28.88 ± 1.98		
304	1.11 ± 0.04	1.03 ± 0.05	0.99 ± 0.07	−29.09 ± 2.11	−18.78 ± 1.1.37	33.92 ± 3.04
310	1.00 ± 0.04	0.85 ± 0.05	1.03 ± 0.03	−29.25 ± 2.21		

The best fit was achieved through tail-fitting with three exponentials and the confidence of each fit was assessed by *χ*^2^ values. The decay components are listed in Table 2, where the *τ*_avg_ of free HSA is 5.586 s. Whereas, the *τ*_avg_ changed to 5.617 and 5.680 s at two different CR concentrations. Evidently, the system formation has hardly any effect on the decay time. Hence, the fluorescence quenching of HSA induced by CR was substantially a static mechanism owning to the ground state CR–HSA compound formation. This finding was in good agreement with the fluorescence quenching inference.

**Table 2:** Fluorescence lifetime decay of 2 µM HSA at different concentrations of CR.

Sample	*τ*_1_ (ns)	*τ*_2_ (ns)	*τ*_3_ (ns)	*α*_1_	*α*_2_	*α*_3_	*τ* (ns)	*χ* ^2^
Free HSA	3.09 ± 0.23	4.72 ± 0.19	6.83 ± 0.36	0.31 ± 0.03	0.04 ± 0.01	0.65 ± 0.03	5.59 ± 0.02	1.09
HSA:CR = 1:1.5	3.22 ± 0.18	5.29 ± 0.22	6.98 ± 0.27	0.34 ± 0.03	0.05 ± 0.01	0.61 ± 0.05	5.63 ± 0.02	1.12
HSA:CR = 1:3.0	3.23 ± 0.15	5.32 ± 0.26	6.96 ± 0.31	0.33 ± 0.02	0.03 ± 0.01	0.64 ± 0.02	5.68 ± 0.01	1.14

### Assessment of binding affinity and force

As noted above, the binding constant (*K*_a_) of static quenching can be calculated from fluorescence spectroscopic data using the modified Stern–Volmer equation^30^:

3 $$\mathrm{log}({F}_{0}-F)/F=\,\mathrm{log}\,{K}_{a}+n\,\mathrm{log}[Q]$$

where *n* refers to the Hill coefficient, which describes the extent of cooperativity during interaction. As listed in Table 1, the values of binding constant (*K*_a_) and *n* in the CR–HSA complex were obtained from the *y*-intercept and slope of the curve (log[(*F*_0_ − *F*)/*F*] versus log[*Q*]) by plotting the double-logarithm regression curve. *K*_a_ was calculated to be (1.14 ± 0.03) × 10^5^ L/mol at 298 K, and the Hill coefficients were equal to one at different temperatures. This finding indicated that CR deserved a single affinity site with moderate binding strength (10^3^–10^6^ L/mol) in HSA. The decrease in *K*_a_ with temperature increasing further proves the formation of CR–HSA complex^31^. This behavior is in good agree with the static quenching mechanism and the results of molecular docking.

In substrate binding, the thermodynamic parameters are allowed to determine binding energy for individual active sites^32^. Based on the research experience of interaction, the binding forces between ligands and biomacromolecules may include multiple hydrophobic, hydrogen bonding, electrostatic and van der Waal’s interaction^33^. To elucidate the interaction forces of CR in HSA, the van’t Hoff equation (Eq. (4)) and Gibbs function (Eq. (5)) was used to confirm thermodynamic constants (enthalpy change: ∆*H*, entropy change: ∆*S*, and change in Gibbs free-energy: ∆*G*)^34^:

4 $${\rm{\Delta }}G=-\,RT\,\mathrm{ln}\,{K}_{{\rm{a}}}$$

5 $${\rm{\Delta }}G={\rm{\Delta }}H-T{\rm{\Delta }}S$$

where *K*_a_ is the same as above obtained and *R* is 8.314 J/mol/K. The values of ∆*H* and ∆*S* were calculated by plotting log*K*_a_ against 1/*T* (Table 1). A negative ∆H indicates that the CR binding process to HSA is exothermic, while the positive contribution to ∆*S* accompanying the freedom loss of translational and rotational atom is associated with process that CR molecular involved into the HSA cavity by positive contributions to the entropy from hydrophobic and electrostatic effects^35^, which is in accordance with the docking results. Moreover, negative ∆*G* values demonstrated the spontaneity of the binding process^36^, marking the spontaneous formation of the CR–HSA complex.

### Analysis of site marker competition

As described in the molecular docking prediction, CR is adjusted in the hydrophobic cavity of site I in HSA. To confirm the binding site, competition experiments were conducted at 298 K using the probes of warfarin sodium, ibuprofen, dansyl-L-arginine, and dansyl-L-sarcosine. Warfarin sodium and ibuprofen were used as specific markers for sites I and II, respectively^37,38^. Dansyl-L-arginine on the FA7 site of HSA also preferentially binds to site I, whereas dansyl-L-sarcosine on FA1 binds to site III (heme-binding site)^22^. With these probes, the displacement of CR to HSA on percentage can be determined as follows^39^:

6 $$I=F/{F}_{0}$$

where *F* and *F*_0_ are the fluorescence intensities of the CR–HSA complex in absent and present of site probe, respectively. The sketch of *F/F*_0_ against different probe concentrations was fitted as shown in Fig. 2b. The intensity of the system was evidently decreased with the addition of warfarin sodium and dansyl-L-arginine. As site I overlaps with FA7 with low affinity for fatty acids, the proportion change of fluorescence intensity was stronger with the addition of warfarin sodium than with dansyl-L-arginine. However, a minimal effect was observed with ibuprofen. Notably, when dansyl-L-sarcosine was added, the combination of CR and HSA was initially decreased without significant changes with increasing marker concentration. This was probably the result of a cooperative binding between CR and dansyl-L-sarcosine to HSA at different sites. Therefore, in these conditions, CR should effectively bind to site I on free HSA.

### Conformational changes of HSA upon the addition of CR

In the binding system, the intermolecular along with intramolecular forces are changing, thereby resulting conformational changes of HSA in the secondary or tertiary. To evaluate the conformation transitions, CD spectroscopy and synchronous fluorescence were performed.

**Figure 3:**
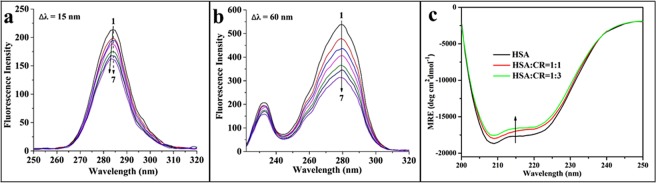
Synchronous fluorescence spectra of HSA in the presence of varying concentrations of CR at (**a**) ∆*λ* = 15 nm and (**b**) ∆*λ* = 60 nm. The direction of the arrow indicates the increase in CR concentration. (**c**) CD spectra of free HSA and HSA complexed with CR at pH 7.4. *c*(HSA) = 2 µM. The molar ratios of HSA to CR were 1:0, 1:1, and 1:3 from bottom to top.

Primarily, specific spectral intervals (Δλ) in synchronous fluorescence were selected at 15 nm and 60 nm to provided characteristic information about the molecular micro-environment of tyrosine and tryptophan residues^40^. The spectra of HSA upon adding different concentrations of CR are shown in Fig. 3(a,b). CR caused a blue shift when Δλ = 15 nm in the *λ*_max_, whereas the peak had no noticeably shift around the *λ*_max_ when Δλ = 60 nm. It indicated that CR binding decreased the polarity and the hydrophobicity of the microenvironment around Tyr and might be located more closely to Tyr than the Trp residue.

Further evidence of the conformational changes of HSA upon the addition of CR was obtained through CD spectroscopy. CD is a recognized technique to detect rapidly for the protein secondary structural changes caused by ligand addition. Figure 3c depicts the CD spectra of HSA in the presence and absence of CR. Two characteristic peaks at 208 and 220 nm are attributed to the *α*-helix conformation, which refer to π → π* and n → π* transition, respectively^41^. When a certain concentration of CR added, the intensity of the peaks decreased without shifting, and the *α*-helical content at 208 nm was determined using the following equation^42^:

7 $$\alpha -\mathrm{helix}\,( \% )=\frac{-MR{E}_{208}-4000}{33000-4000}\times 100$$

where 4,000 and 33,000 are the mean residue ellipticity (MRE) values of the random coil conformation and pure *α*-helix at 208 nm. Similar signature of free HSA and CR–HSA complex in the CD spectra was observed involving the position and shape of peaks. The percentage (52.08%) of the *α*-helical content for free HSA dropped to 49.25% and 47.69% with a molar concentration ratio at 1:1 and 1:3 (HSA:CR), respectively. The above analytical results indicated that CR resulted in a modicum of unfolding in the constituent polypeptides and induced a slight effect on the HSA secondary structure, suggesting that the function of HSA was not disturbed. These results were in line with computer simulations.

## Conclusions

In present study, a detailed investigation on the binding properties between food CR and HSA by computational and experimental methods. The YASARA strategy-based blind docking simulation and MD predicted the optimal binding site of CR located in the hydrophobic cavity of site I. Fluorescence quenching constant and constancy in a lifetime values revealed that the binding process was involved by a static quenching mechanism. CR bound to HSA with a moderate binding affinity and was driven primarily by electrostatic interaction force. The combination of the HSA–CR complex decreased the content of *α*-helix structures and the hydrophobic microenvironment around Tyr in HSA. The results of multi-spectroscopic and computational methods supplemented and thereby reinforced each other. Overall, given the wide range of food colorants, further studies should be conducted to understand the organic molecule transportation and bioaccumulative toxicity in the human body.

## Materials and Methods

### Reagents and Chemicals

Fatty-acid-free HSA (A1887-5G), dansyl-L-arginine, and dansyl-L-sarcosine were procured from Sigma-Aldrich (St. Louis, USA). CR (CAS: 6358-53-8, 98% purity). warfarin sodium, and ibuprofen were purchased from J&K Scientific Ltd. (Beijing, China). All other materials and reagents were of analytical grade. The HSA stock solution was prepared at 20 µM concentration in 50 mM phosphate-buffered saline (PBS) solution (pH 7.4, containing 0.1 M NaCl), which was measured by its UV-Vis absorption using an extinction coefficient of 36,850 mol^−1^ cm^−1^ at 280 nm^12^. The concentration of CR stock solution was 4 mM in ethanol. All sample solutions stored at 4 °C in the dark. Ultrapure water was used throughout this work.

### Computational methods

#### Molecular docking simulations

As automated docking is highly advantageous to predict the configurations of interaction system, YASARA v17.4.17 program based on VINA module was selected to analyse the CR–HSA complexes. The available structures of free HSA (receptor 1, PDB ID: 1AO6), HSA complexed with heme (receptor 2, PDB ID: 1N5U), HSA complexed with myristic acid and hemin (receptor 3, PDB ID: 1O9X), and CR (ligand, CID: 9570225) were gathered from the RCSB Protein Data Bank and PubChem. Pre-docking, all water molecules in receptors were eliminated and pH 7.4 was performed in the docking preparation. The receptors and ligand were optimized by the energy minimization module in YASARA platform. A fully flexible docking algorithm on receptors was applied in the simulation process, in a similar manner as the ligand, to obtain all potential theoretically reasonable binding modes. The grid box size was set 1.5 times the volume of HSA and contained all atoms with 100 docking runs. Docking protocol was conducted to the processed receptor and ligand structures. The minimum energy docked conformation was determined at different conformations, and the optimal binding energy conformation was selected for further analysis. The brief 2D interaction map was generated using the LIGPLOT program.

#### MD simulation

YASARA v16.7.22 package with AMBER14 force field was applied for MD simulation. The CR–HSA complex with the best binding energy was selected for further assess the stability of the system structure. Firstly, the partial atomic charges of CR were optimized via AM1-BCC model 20^43^, and then the energy of the CR–receptor conformation was computed. Second, a general heating step was followed with temperature set at 298 K, and the protonation state of ionizable residues was arranged at pH 7.4. Counter ions were added randomly with replacing water molecules by Na^+^ or Cl^−^ to provide a charge-neutral system. To simulate the long-range Coulomb interactions with a cut-off of 8.0 Å, Particle Mesh Ewald summation was performed here. Using predefined macros^44^, data were collected to record trajectories per 10 ps. Multiple time steps of intramolecular (1.25 fs) and intermolecular (2.5 fs) forces were simulated, respectively.

### Fluorescence spectroscopy measurements

Steady-state fluorescence quenching spectra were recorded on Varian Cary Eclipse fluorescence spectrophotometer (Santa Clara, CA, USA) equipped with a 1.0 cm quartz cell. Fluorometric experiments were carried out with a fixed 2.0 µM HSA concentration and serially increased CR concentrations (0, 1, 2, 3, 4, 5, and 6 µM) at 298 K, 304 K, and 310 K, respectively. In addition, the UV–vis spectra of CR (6 µM) and the CR–HSA complex are shown in Fig. S1. All solutions were recorded within wavelength range of 300 nm to 500 nm under 280 nm excitation wavelength. Excitation/emission slit widths were adjusted to 5/10 nm. The effect of the inner filter here was eliminated as fellow:

8 $${F}_{corr}={F}_{obs}\times {e}^{\frac{{A}_{ex}+{A}_{em}}{2}}$$

where *F*_*corr*_ and *F*_*obs*_ are the corrected and observed fluorescence intensities, respectively. *A*_*ex*_ and *A*_*em*_ refer to the absorbance values of HSA under 280 and 338 nm wavelengths, respectively.

Synchronous fluorescence spectra of HSA varying TF were recorded under 15 or 60 nm (Δ*λ* = *λ*_em_−*λ*_ex_), and the other parameter settings were the same as those above.

### Time-resolved fluorescence measurements

The time-correlated single-photon fluorescence spectra were conducted using the HORIBA Jobin Yvon FluoroLog-TCSPC spectrofluorometer (HORIBA, Les Ulis, France) at *λ*_ex_ = 280 nm and *λ*_em_=345 nm at 298 K. Free HSA (2.0 µM) and CR–HSA complex solutions (at ratio 1:0, 1:1.5, 1:3.0, [HSA]:[CR]) were tested. The tail-fitting method was applied to analyze the fluorescence lifetime data, and the quality of each fitting was judged by the typical *x*^2^ values and residuals.

### Site competition experiments

Site probe competition studies were tested using warfarin sodium and ibuprofen. The ratio of CR to HSA was maintained at 1:1 to minimize non-specific binding of probes. The complex mixture solution was added into volumetric flasks. After 1 h of incubation, warfarin, ibuprofen, dansyl-L-arginine, and dansyl-L-sarcosine were gradually added to the complex (with CR molar ratio ranging from 0.5 to 3.0). Fluorescence data were collected under the same experimental conditions stated in the preceding fluorescence titration experiment.

### CD spectroscopy measurements

An automatic recording spectrophotometer (Model 400, AVIV, USA) was applied to record the CD spectra of CR on HSA at 298 K. Peltier temperature control device was equipped in a 10 mm cell in nitrogen atmosphere. The spectra of HSA (2.0 µM) in 10 mM phosphate buffer saline solution in absent and present CR (at ratio of 1:0, 1:1, 1:3) were obtained in the range of 190–260 nm, with a 200 nm/min scanning speed. CD results were expressed as the MRE and defined using the following equation^42^:

9 $$MRE=\frac{Intensity\,of\,CD\,(mdeg)\,}{{C}_{p}nl\times 10}$$

where *C*_*P*_ is the HSA concentration at 208 nm; *n* is 585 for the number of HSA residues; and *l* is 1 cm for the path length, MRE is in in deg cm^2^ dmol^−1^.

### Statistical analysis

Data were expressed as the mean ± standard deviation in the binding analysis. Statistical comparisons between the means of individual groups were performed using one-way analysis of variance. All assays were conducted in triplicate.

## Supplementary information

Supporting information
