# Supplementary material for: Computational and spectroscopic analysis of interaction between food colorant citrus red 2 and human serum albumin
Source: Sci Rep. 2019 Feb 7;9:1615. doi: 10.1038/s41598-018-38240-9 (PMC6367341; doi:10.1038/s41598-018-38240-9)
Supplement: Supplementary file 1 — Supporting information [file 41598_2018_38240_MOESM1_ESM.docx]

**Computational and spectroscopic analysis of interaction between food colorant citrus red 2 and human serum albumin**

Di Wu*, Jinqiu Wang, Dayu Liu, Yin Zhang, Xia Hu

Key Laboratory of Meat Processing of Sichuan, College of Pharmacy and Biological Engineering, Chengdu University, Chengdu 610106, China

**Corresponding author：**Dr. Di Wu, Key Laboratory of Meat Processing of Sichuan, College of Pharmacy and Biological Engineering, Chengdu University, Chengdu 610106, China.

**E-mail address:** diwulab@163.com


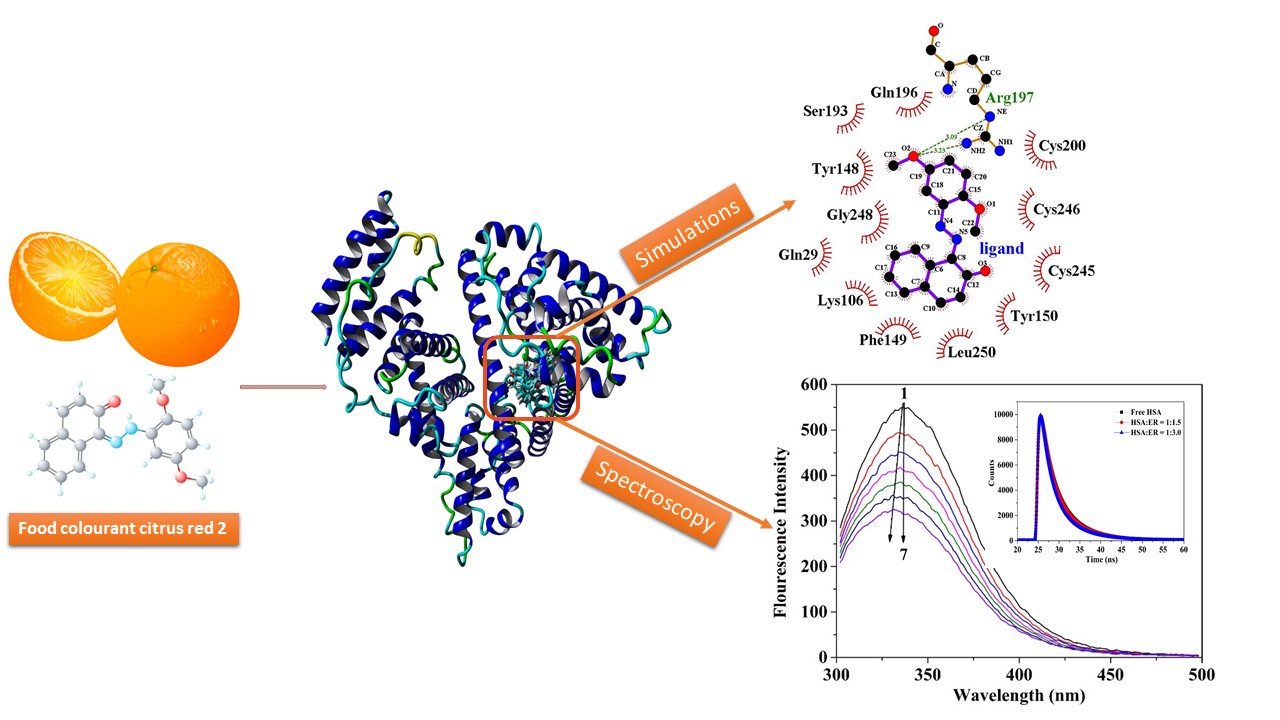


Graphic abstract: Prospective binding analysis of citrus red 2 with human serum albumin





Figure S1. The UV-vis absorbance spectra of 6 µM CR and of CR-HSA complex ([CR]:[HSA]=3:1).
